# Longitudinal Associations of Leptin and Adiponectin with Heart Rate Variability in Children

**DOI:** 10.3389/fphys.2017.00498

**Published:** 2017-07-12

**Authors:** Roos Van De Wielle, Nathalie Michels

**Affiliations:** Department of Public Health, Faculty of Medicine and Health Sciences, Ghent University Ghent, Belgium

**Keywords:** leptin, adiponectin, heart rate variability, children, longitudinal, autonomic nervous system

## Abstract

For early prevention of cardiovascular disease, early detection and risk factor insights are necessary. The autonomic balance reflects cardiovascular risk and can be measured by heart rate variability (HRV). Therefore, our purpose is to examine associations between HRV and the energy-related biomarkers leptin and adiponectin in children. Participants of this study were Belgian children recruited for the longitudinal ChiBS study (year 2010–2012). HRV was measured and fasting blood samples were taken in 249 children at baseline (4.4–11.0 y) and 223 children at follow-up (6.7–12.2 y). Cross-sectional and longitudinal linear regression analyses were separated by sex and adjusted for age, socio-economic status, body fat%, negative emotions, puberty, and mean heart rate. Leptin was a negative cross-sectional and longitudinal predictor of parasympathetic activity in boys; while leptin in girls was cross-sectionally associated with higher LF and LF/HF suggesting sympathetic predominance. Adiponectin was a negative cross-sectional and longitudinal predictor of parasympathetic activity in boys; but when adjusting for mean heart rate, this effect disappeared and adiponectin was a positive cross-sectional and longitudinal predictor of parasympathetic activity in girls. These results stress the importance of considering sex differences and adjustment for heart rate in testing HRV predictors. Leptin seemed disadvantageous for the autonomic balance, while adiponectin seemed advantageous for the autonomic balance in girls only. More research is needed to see whether leptin and adiponectin are interesting in cardiovascular screening/prevention or in determining the cardiovascular gain during weight loss follow-up.

## Introduction

Cardiovascular diseases cause invalidity, diminish life quality and are a main cause of death in the Western world (World Health Organization, 2012). Several studies confirm that cardiovascular problems are initiated in childhood. Consequently, prevention of cardiovascular disease should start at an early age. Herein, a good understanding of the pathogenesis and risk factors is necessary, and the early detection of problems is important. In this context, cardiovascular problems can be non-invasively reflected by the autonomic balance. A dysfunctional autonomic balance, i.e., an increase in sympathetic activity and/or a decrease in parasympathetic activity, is related to cardiovascular diseases and cardiovascular mortality (Baum et al., 2013).

Since cardiovascular disease is influenced by overweight and obesity, it seems straightforward to define biomarkers related with overweight and cardiovascular health in the search of pathogenic markers (Giannini et al., 2009; Baum et al., 2013; Mahmood et al., 2014). Overweight and obesity are associated with hyperplasia and hypertrophy of adipose tissue, which functions as an endocrine organ. After all, adipose tissue is responsible for the secretion of more than 50 hormones and signal molecules which are involved in energy homeostasis and inflammation and which are collectively referred to as adipokines. In the perspective of obesity and cardiovascular disease, the predictive power of the 16 kDa adipokine leptin and the 30 kDa cardioprotective adiponectin seems most relevant to study (Bruyndonckx et al., 2013).

Leptin is involved in energy homeostasis since it suppresses the appetite, stimulates thermogenesis, increases fatty acid oxidation, reduces plasma glucose, and reduces body fat. People with overweight and obesity have higher leptin concentrations but often become leptin resistant. This is a phenomenon whereby the increased leptin concentrations can no longer suppress appetite. The high leptin concentrations in overweight and obese people are related with the pathological changes of the vasculature in these patients: leptin is atherogenic (Harwood, 2012; Bruyndonckx et al., 2013; Charles et al., 2015).

Adiponectin is cardioprotective as it has anti-inflammatory and anti-atherogenic characteristics. It decreases the production of inflammatory cytokines, vascular cell adhesion molecules, and foam cells and induces a higher production of nitric oxide. In addition, adiponectin increases insulin sensitivity. Adiponectin is increasingly used as a biomarker of insulin sensitivity or as predictor of cardiovascular risk, since life style changes can easily influence the adiponectin concentrations in a positive way (Harwood, 2012; Caselli et al., 2013).

In this study, the association between the autonomic balance, measured by heart rate variability (HRV), and the adipokines leptin, and adiponectin is examined in healthy children. Until now, those associations have been mainly explored in adults, and most often in clinical populations. In several clinical adult populations, a negative association between adiponectin and sympathetic activity was seen, as well as a positive association with parasympathetic activity (Fasshauer et al., 2003; Takahashi et al., 2007; Boer-Martins et al., 2011; Barbosa-Ferreira et al., 2015). However, literature does not report unambiguously on the association between the autonomic balance and leptin. A positive association between leptin and sympathetic activity is suggested by some authors (Paolisso et al., 2000; Flanagan et al., 2007; Pieterse et al., 2014). Other studies revealed a negative association with sympathetic activity (Charles et al., 2015) and parasympathetic activity (Paolisso et al., 2000; Flanagan et al., 2007). In this study, we hypothesize that high leptin concentrations might be related to increased sympathetic activity, whereas adiponectin might influence the autonomic balance in favor of parasympathetic activity.

## Materials and methods

### Design

Participants of this study were Belgian children recruited for the longitudinal ChiBS study (year 2010 and 2012). Children were between 4.4 and 11.0 years old at baseline and between 6.7 and 12.2 years old at follow-up. More details on the ChiBS study and its measurements have been described elsewhere (Michels et al., 2012). Since blood withdrawal was optional, not all participant could be included in the current analysis. In 2010, 270 from the 520 children had information on all variables for the current analyses: serum values and HRV but this number was diminished to 249 after checking all confounders. Of them, 230 also had information on HRV and serum values in 2012 but this number was diminished to 223 after checking all confounders. On the examination day in 2010 and 2012, fasting blood withdrawal was executed (i.a. for leptin and adiponectin level determination) and HRV was measured. The study was conducted according to the guidelines laid down in the Declaration of Helsinki and the project protocol was approved by the Ethics Committee of the Ghent University Hospital. A written informed consent was obtained from the parents and a verbal assent from the children.

### Biomarkers: leptin and adiponectin

Leptin and adiponectin were measured using a venous blood sample after an overnight fast. The venipuncture was done at the level of the antecubital vein in the left arm. Serum tubes were stored at room temperature for 30 min to allow clotting. Processing of the blood samples was performed within 4 h after collection. All blood samples were centrifuged at 2,500 g for 10 min and were stored at −80°C until further analyses of the extracted serum. Leptin serum concentrations were measured in 2010 using a Meso Scale Discovery sandwich electrochemiluminescence immunoassay (inter-assay CV 2.4% for low, 3.9% for high controls; intra-assay CV 2.7% for low, 5.1% for high controls) and in 2012 using a Millipore radioimmunoassay in a certified laboratory (inter-assay CV 3.0% for low, 6.2% for high controls; intra-assay CV 3.4% for low, 8.3% for high controls). Millipore radioimmunoassay was also used to measure adiponectin serum concentrations, in both 2010 and 2012.

### HRV

To define HRV, each child was individually examined in a quiet room in supine position (i.e., lying down with the face up) during 10 min. Children were asked to refrain from strenuous physical activity on the measurement day. The child was encouraged to be calm, to breath normally and not to speak or move during the 10 min of HRV measurement. The heart rate belt was fixed around the chest and measurements were started after a couple of minutes when the signal was stabilized. RR-intervals (RRI) were recorded at a sampling rate of 1,000 Hz with the elastic electrode belt Polar Wear link 31 using a Wind link infrared computer transmitter. This low-cost device has a proven validity in supine position compared to the gold standard of an electrocardiogram device (*r* > 0.97; Gamelin et al., 2006), also in children (*r* > 0.99; Gamelin et al., 2008).

The middle 5 min were manually checked on their quality and if necessary, another appropriate 5 min interval was chosen. Quality was defined as no large RRI outliers, an equidistance between consecutive RRI points and unimodal and Gaussians RRI and heart rate distribution graphics. As such, disturbing phenomena like the Valsalva maneuvre were excluded. Data processing to obtain time-domain and frequency-domain parameters was performed with the free, professional HRV Analysis Software of the University of Kuopio, Finland (Niskanen et al., 2004). The RR series were de-trended using the Smoothness priors method with alpha = 300 and a cubic interpolation at the default rate of 4 Hz was done (Task Force, 1996). Low frequency (LF) and high frequency (HF) bands were analyzed between 0.04–0.15 and 0.15–0.4 Hz, as default (Task Force, 1996) using the autoregression method. In addition, the LF/HF ratio was calculated as approximate parameter of the sympathovagal balance. HF was expressed in power units and was used as a marker of the parasympathetic activity. LF was expressed in normalized units [=LF/(total power − very low power)] to roughly represent the sympathetic activity (although it might be a mixture of sympathetic and parasympathetic activity). In the time domain analysis, pNN50 (percentage of successive normal sinus RR intervals >50 ms) was used as marker of the parasympathetic activity. In Supplementary Tables 1–3 the results are shown for 4 other HRV parameters that do not give additional information next to the 4 others mentioned above: root mean square of successive differences (RMSSD), standard deviation of normal RR intervals (SDNN), HF in normalized units, LF in power.

### Confounding factors

Sex, age, parental education level, body fat%, negative emotions and mean heart rate were considered as potential confounding factors, based on previous research (Porges et al., 1994; Blum et al., 1997). When analyzing leptin, pubertal status was considered as extra confounding factor in 2012. The children's sex and birth date were reported by the parent. To represent socio-economic status, parental education level was assessed by questionnaire according to the International Standard Classification of Education (Unesco, 2010). Six groups (1 = low income, 6 = high income) were distinguished. Due to the limited percentage of children in the lowest groups, group 1 until 4 and groups 5 and 6 were respectively recoded in “low income” and “high income.” Body fat% was reliably measured using air-displacement plethysmography (BOD POD®) in both 2010 and 2012. Children were measured following standardized procedures: twice in tight-fitting bathing suit with swim cap to rule out air trapped in clothes or hair and child-specific conversion factors were applied (McCrory et al., 1995). For negative emotions, children were questioned about their recent feelings by rating the feelings anger, anxiety, and sadness on a 0 to 10 Likert-scale (0 “not at all” to 10 “very strong;” Zimmer-Gembeck et al., 2009). To help the children understand these distinct feelings, pictures of a social skills training game for very young children were displayed next to the question. The sum of the three negative emotions was used as confounder. Pubertal status was assessed in 2012 using Tanner classification based on staging of pubic hair distribution and genital development for boys, and pubic hair distribution and breast development for girls. Due to the limited percentage of children in the Tanner stages higher than stage I, these data were recoded into two groups (“no sign of puberty” vs. “signs of puberty”). Heart rate was defined in analogy to HRV: in supine position with an electrode band and infrared computer transmission.

### Statistical analyses

Statistical analyses were performed using SPSS 23 (SPSS Inc., Chicago, IL) and all *p* < 0.05 were considered significant. The association between the biomarkers (predictor) and HRV (outcome) was tested with cross-sectional linear regression using 2010 and 2012 variables. When the regression residuals were not normally distributed, outcome variables were transformed by calculating the natural logarithm. Since all variables were available in both 2010 and 2012, the association between adiponectin and leptin (predictors) and HRV (outcome) was also tested with longitudinal mixed model linear regression. This multilevel method enables testing the longitudinal change in the slope (two measurements within the individual) by including predictor and outcome factors of the two time points; thus having the advantage of not using difference scores but slopes for longitudinal analyses. The longitudinal effect is reflected by the time^*^predictor variable (while also including the separate time and predictor variable in the model). Adiponectin was also tested as outcome of HRV, since literature reports unclearly about the role of adiponectin in relationship to autonomic activity.

Adjustment for confounding factors was performed in several consecutive steps. First, we adjusted for age, sex, and parental education as general confounders. Secondly, body fat% was added as additional confounder. Thirdly, negative emotions were added as additional confounder since HRV might also be influenced by stress/emotions (Porges et al., 1994; Michels et al., 2013). In case of leptin, adjustment for pubertal status was included in the analyses of 2012. At last, heart rate was included as additional confounding factor. A stepwise regression model did not show different results compared to this consecutive confounder model. Since sex interaction terms (i.e., sex^*^predictor) were often significant in our sample, analyses were split by sex.

## Results

### Descriptive characteristics

Descriptive values of leptin levels, adiponectin levels, HRV indices, and confounding factors can be found in Tables 1A,B. Some sex differences were detected with girls having significantly higher leptin levels at baseline and at follow-up; while pNN50 and HF at follow-up were significantly higher in boys compared to girls. When comparing data at baseline and at follow up, leptin was significantly increased over time in both sexes, while adiponectin and LF/HF ratio were significantly increased over time in girls only, and LF in boys only. Leptin was positively correlated with age, while LF and LF/HF were negatively correlated with age. At baseline, 6.74% of the boys and 10.13% of the girls were overweight or obese. At follow-up, respectively 6.54 and 12.07% of the children weighed too much. As expected, leptin was positively correlated with body fat% (*r* = 0.448 *p* < 0.001 at baseline; *r* = 0.776 *p* < 0.001 at follow-up) but adiponectin was not.

**Table 1A:** Descriptive statistics of the population.

**2010**				**2012**				**≠ 2010 and 2012**	
**Adipokines**	**♂—*N* = 125**	**♀—*N* = 124**	**≠ ♂ & ♀**	**Adipokines**	**♂—*N* = 107**	**♀—*N* = 116**	**≠ ♂ & ♀**	**♂**	**♀**
	**Median [IQR]**	**Median [IQR]**	***P***		**Median [IQR]**	**Median [IQR]**	***p***	***p***	***p***
Leptin (ng/ml)	1.02 [1.05]	1.70 [3.01]	**<0.001**	Leptine (ng/ml)	2.48 [1.69]	4.29 [3.54]	**<0.001**	**<0.001**	<0.001
Adiponectin (μg/ml)	9.19 [63]	8.56 [13]	0.325	Adiponectine (μg/ml)	14 [5.10]	15 [4.95]	0.073	0.209	**0.006**
**HRV Parameters**	♂	♀	**≠ ♂ & ♀**	**HRV Parameters**	♂	♀	**≠ ♂ & ♀**	♂	♀
pnn50 (%)	41 [25]	41 [29]	0.645	pnn50 (%)	44 [24]	40 [29]	**0.024**	0.526	0.623
lf (normalized units)	47 [21]	47 [17]	0.500	lf (normalized units)	47 [18]	46 [19]	0.986	**0.016**	0.484
hf (ms^∧^2)	411 [508]	422 [505]	0.677	hf (ms^∧^2)	462 [567]	393 [632]	**0.049**	0.152	0.201
lf/hf	1.05 [1.07]	0.98 [0.80]	0.162	lf/hf	1.25 [1.15]	1.19 [1.36]	0.985	0.616	**0.013**

*♂, man; ♀, woman; N, number; [IQR], interquartile range; p, significance; HRV, Heart Rate Variability; pNN50, percentage of successive normal sinus RR intervals >50 ms; LF, low frequency; HF, high frequency; LF/HF, low frequency/high frequency. Bold indicates p < 0.05*.

**Table 1B:** Descriptive statistics of the confounding factors.

**2010**			**2012**		
**Confounding factors**	**♂ *N* = 125**	**♀ *N* = 124**	**Confounding factors**	**♂ *N* = 107**	**♀ *N* = 116**
	**% Median [IQR]**			**% Median [IQR]**	**% Median [IQR]**
Age	8.00 [2.00]	8.40 [1.60]	Age	9.79 [2.30]	10.06 [2.20]
Body fat (%)	18 [6.17]	21 [9.85]	Body fat%	16 [7.60]	23 [8.78]
Mean heart rate (beats/min)	79 [13]	82 [9.71]	Mean heart rate (beats/min)	75 [15]	79 [14]
Negative emotions (0–30)	6.00 [8.00]	7.50 [7.00]	Negative emotions (0–30)	4.00 [6.00]	5.00 [7.00]
ses (low/high)	23.20% low 76.80% high	22.58% low 77.42% high	Tanner stadium (no signs/puberty signs)	76.64% no signs 23.36% puberty signs	66.38% no signs 33.62% puberty signs

*♂, man; ♀, woman; N, number; [IQR], interquartile range; SES, socioeconomic status*.

### Associations of leptin with the autonomic balance

Results of leptin as predictor of the autonomic balance at baseline are shown in Table 2. In girls, a positive significant association was found for leptin with LF and LF/HF, also after adjustment for confounding factors. At follow-up (see Table 3), leptin was negatively associated with pNN50 and HF in boys. After adjustment for body fat%, the significance for HF disappeared, but the association with pNN50 remained all the way. Longitudinally, a negative significant effect of leptin on pNN50 and HF was detected in boys (Table 4).

**Table 2:** Linear regression with adipokines predicting heart rate variability, 2010 (*N* = 249).

	**♀/♂**	**pnn50**			**LFnormalized**			**HFpower**			**LF/HF**		
		**adjR^2^**	**β**	***p***	**adjR^2^**	**β**	***p***	**adjR^2^**	**β**	***p***	**adjR^2^**	**β**	***p***
**Leptin**	♂	−0.005	0.055	0.540	−0.008	0.003	0.971	−0.008	−0.015	0.868	−0.004	−0.064	0.478
	♀	−0.008	0.018	0.840	0.013	0.144	0.110	−0.007	−0.028	0.759	0.014	0.149	0.100
Model 1	♂	0.010	0.026	0.777	−0.014	0.020	0.824	−0.005	−0.032	0.727	−0.002	−0.045	0.625
	♀	−0.012	0.007	0.942	0.014	0.171	0.064	−0.014	−0.036	0.694	0.021	0.178	0.052
Model 2	♂	0.004	0.032	0.724	−0.020	0.014	0.879	0.012	−0.012	0.893	−0.004	−0.054	0.557
	♀	−0.013	0.100	0.455	0.033	0.342	**0.010**	−0.021	0.007	0.958	0.052	0.384	**0.004**
Model 3	♂	−0.001	0.033	0.723	−0.022	0.014	0.883	0.005	−0.012	0.895	−0.011	−0.054	0.557
	♀	0.074	0.160	0.215	0.067	0.302	**0.021**	0.125	0.084	0.505	0.105	0.336	**0.009**
Model 4	♂	0.498	−0.065	0.327	0.003	0.039	0.676	0.335	−0.091	0.228	0.050	−0.018	0.838
	♀	0.556	−0.052	0.567	0.219	0.424	**0.001**	0.484	−0.100	0.310	0.245	0.453	**<0.001**
**Adiponectin**	♂	−0.007	−0.026	0.769	−0.006	−0.045	0.615	−0.008	0.018	0.840	0.001	−0.089	0.325
	♀	−0.003	0.074	0.417	−0.008	0.024	0.793	−0.006	0.047	0.608	−0.008	−0.026	0.775
Model 1	♂	0.011	−0.045	0.619	−0.013	−0.035	0.699	−0.006	0.015	0.868	0.003	−0.082	0.363
	♀	−0.007	0.077	0.399	−0.014	0.030	0.746	−0.013	0.051	0.577	−0.010	−0.016	0.857
Model 2	♂	0.005	−0.042	0.643	−0.018	−0.039	0.673	0.013	0.025	0.782	0.001	−0.087	0.339
	♀	−0.012	0.077	0.400	−0.023	0.030	0.748	−0.018	0.051	0.578	−0.018	−0.016	0.858
Model 3	♂	0.001	−0.035	0.706	−0.020	−0.051	0.579	0.006	0.031	0.738	−0.005	−0.096	0.299
	♀	0.070	0.089	0.310	0.024	0.020	0.822	0.127	0.067	0.433	0.052	−0.028	0.755
Model 4	♂	0.494	0.030	0.646	0.006	−0.068	0.457	0.334	0.084	0.268	0.064	−0.121	0.176
	♀	0.562	0.083	0.168	0.137	0.023	0.784	0.483	0.062	0.346	0.152	−0.025	0.766

*♂, man; ♀, woman; N, number; pNN50, percentage of successive normal sinus RR intervals >50 ms; LF, low frequency; HF, high frequency; LF/HF, low frequency/high frequency; ADJR^2^, adjusted r^2^; β, standardized regression coefficient; p, significance—Model 1, correction for age, socioeconomic status; Model 2, Model 1 + body fat%; Model 3, Model 2 + negative emotions; Model 4, Model 3 + mean heart rate. Bold indicates p < 0.05*.

**Table 3:** Linear regression with adipokines predicting heart rate variability, 2012 (*N* = 223).

	**♀/♂**	**Pnn50**			**LFnormalized**			**HFpower**			**LF/HF**		
		**adjR^2^**	**β**	***p***	**adjR^2^**	**β**	***p***	**adjR^2^**	**β**	***p***	**adjR^2^**	**β**	***p***
**Leptin**	♂	0.145	−0.392	**<0.001**	−0.006	0.062	0.526	0.067	−0.276	**0.004**	−0.009	0.005	0.958
	♀	−0.005	−0.057	0.542	−0.009	−0.009	0.920	−0.008	−0.029	0.754	−0.008	−0.018	0.849
Model 1	♂	0.144	−0.423	**<0.001**	0.020	0.115	0.255	0.087	−0.325	**0.001**	0.046	0.074	0.459
	♀	−0.019	−0.074	0.451	−0.012	0.019	0.844	−0.026	−0.035	0.725	−0.017	0.005	0.961
Model 2	♂	0.135	−0.435	**0.002**	0.015	0.043	0.772	0.086	−0.232	0.103	0.043	−0.014	0.925
	♀	0.001	0.139	0.368	−0.019	−0.034	0.827	0.017	0.248	0.107	−0.024	−0.057	0.713
Model 3	♂	0.142	−0.408	**0.004**	0.006	0.050	0.738	0.077	−0.228	0.116	0.036	−0.004	0.979
	♀	−0.006	0.137	0.377	0.001	−0.027	0.863	−0.008	0.247	0.110	−0.014	−0.051	0.741
Model 4	♂	0.133	−0.407	**0.004**	0.021	0.042	0.775	0.069	−0.226	0.120	0.063	−0.013	0.929
	♀	0.012	0.125	0.415	−0.008	−0.029	0.850	0.015	0.239	0.122	−0.019	−0.056	0.719
Model 5′	♂	0.557	−0.210	**0.041**	0.056	−0.024	0.869	0.452	−0.039	0.730	0.086	−0.078	0.592
	♀	0.637	0.061	0.514	0.145	0.010	0.942	0.474	0.182	0.106	0.129	−0.015	0.920
**Adiponectin**	♂	0.024	−0.182	0.060	−0.002	0.086	0.379	0.071	−0.283	**0.003**	0.001	0.098	0.314
	♀	−0.009	0.013	0.894	−0.007	−0.042	0.658	−0.004	0.070	0.457	−0.007	−0.039	0.678
Model 1	♂	0.006	−0.185	0.064	0.017	0.098	0.319	0.073	−0.292	**0.003**	0.054	0.113	0.242
	♀	−0.024	0.018	0.851	−0.009	−0.053	0.577	−0.022	0.072	0.457	−0.015	−0.048	0.614
Model 2	♂	0.073	−0.155	0.105	0.022	0.086	0.384	0.133	−0.266	**0.005**	0.054	0.103	0.289
	♀	−0.006	0.009	0.925	−0.017	−0.051	0.595	−0.003	0.062	0.514	−0.023	−0.047	0.629
Model 3	♂	0.101	−0.187	0.053	0.012	0.084	0.405	0.132	−0.279	**0.004**	0.045	0.098	0.320
	♀	−0.013	0.015	0.880	0.004	−0.069	0.470	−0.011	0.066	0.495	−0.012	−0.062	0.524
Model 5	♂	0.538	−0.014	0.840	0.056	0.024	0.816	0.467	−0.128	0.096	0.085	0.041	0.682
	♀	0.647	0.106	0.066	0.158	−0.115	0.197	0.483	0.145	**0.038**	0.140	−0.107	0.235

*♂, man; ♀, woman; N, number; pNN50, percentage of successive normal sinus RR intervals >50 ms; LF, low frequency; HF, high frequency; LF/HF, low frequency/high frequency; ADJR^2^, adjusted r^2^; β, standardized regression coefficient; p, significance; Model 1, correction for age, socioeconomic status; Model 2, Model 1 + body fat%; Model 3, Model 2 + negative emotions; Model 4, Model 3 + pubertal status (only relevant for leptin); Model 5′ (for leptin), Model 4 + mean heart rate; Model 5, Model 3 + mean heart rate. Bold indicates p < 0.05*.

**Table 4:** Mixed model regression, longitudinally.

	**♀/♂**	**pnn50**		**LFnormalized**		**HFpower**		**LF/HF**	
		***b***	***p***	***b***	***p***	***b***	***p***	***b***	***p***
**LEPTIN**									
Model 1	♂	−2.857	**<0.001**	0.378	0.525	−0.035	**0.028**	0.002	0.893
	♀	−0.259	0.563	−0.359	0.344	0.002	0.884	0.008	0.346
Model 2	♂	−2.702	**0.001**	0.139	0.839	−0.015	0.420	−0.006	0.687
	♀	−0.142	0.753	−0.272	0.481	0.001	0.896	−0.006	0.490
Model 3	♂	−1.607	**0.016**	−0.257	0.732	0.012	0.473	−0.016	0.292
	♀	0.140	0.614	0.055	0.893	0.002	0.799	0.002	0.798
**ADIPONECTIN**									
Model 1	♂	−0.798	**0.023**	0.306	0.297	−0.023	**0.002**	0.006	0.304
	♀	0.114	0.786	−0.122	0.718	0.011	0.256	−0.003	0.681
Model 2	♂	−0.711	**0.039**	0.279	0.343	−0.021	**0.005**	0.006	0.347
	♀	0.085	0.838	−0.120	0.722	0.011	0.277	−0.003	0.685
Model 3	♂	0.023	0.928	0.123	0.674	−0.007	0.232	0.001	0.803
	♀	0.323	0.154	−0.249	0.431	0.017	**0.015**	−0.006	0.424

♂, man; ♀, woman; N, number; pNN50, percentage of successive normal sinus RR intervals >50 ms; LF, low frequency; HF, high frequency; LF/HF, low frequency/high frequency; b, regression coefficient of the time^*^predictor parameter to show the longitudinal relation—p, significance; Model 1, correction for age, socioeconomic status; Model 2, Model 1 + body fat%; Model 3, Model 2 + mean heart rate. Bold indicates p < 0.05.

### Association of adiponectin with the autonomic balance

As seen in Table 2, there were no significant associations between adiponectin and HRV at baseline. At follow up (see Table 3), a negative association between adiponectin and HF was seen in boys, which disappeared after adjusting for mean heart rate. However, significant associations in girls were seen after adjustment for mean heart rate: positive association with HF and borderline significant with pNN50. Longitudinal analyses (Table 4) showed similar results as the 2012 cross-sectional analyses. The negative effect on pNN50 and HF in boys disappeared after adjustment for mean heart rate. In girls, a positive effect of adiponectin on HF was visible after adjustment for mean heart rate. The analyses considering adiponectin as outcome of HRV (data not shown), did not show any significant results.

## Discussion

In a group of Belgian primary school children, we aimed to examine the cross-sectional and 2-year longitudinal associations between the adipokines leptin and adiponectin and the autonomic balance as measured by HRV. The associations differed between boys and girls and the tested confounders seemed to have an important role in the associations. This study confirmed that leptin is positively associated with sympathetic activity, but only in girls and cross-sectionally. Besides, high leptin concentration was a cross-sectional and longitudinal predictor of decreased parasympathetic activity, mainly in boys. The hypothesis of adiponectin as a positive predictor of parasympathetic activity was also confirmed in this cross-sectional and longitudinal study, but only in girls and after adjustment for heart rate. Contrary, adiponectin was a negative predictor of parasympathetic activity in boys but no longer after adjustment for heart rate.

### Association of leptin and the autonomic balance

In girls, there was a positive association of leptin with LF/HF and LF at baseline, roughly representing higher sympathetic activity. Interestingly, the association was stronger after correction for the tested confounding factors (e.g., from β = 0.384 to β = 0.453 for LF/HF), with the biggest impact after adjusting for body fat% and mean heart rate. The confounding role of body fat% seems straightforward as leptin is produced by adipose tissue increasingly with bigger or more fat cells (Fried et al., 2000) and as weight decrease has previously been related to HRV improvements probably due to changed free fatty acid levels (Li et al., 2009). Those findings also imply the importance of so-called negative confounders: confounders that cause an underestimation of the association. For example, age was negatively correlated with LF and LF/HF in this study and significantly positively with leptin.

The positive association between leptin and LF or LF/HF in girls is analogous to the findings of Flanagan et al. who showed a positive association in adult women between leptin and sympathetic activity (Flanagan et al., 2007), although others found a positive association between leptin and sympathetic activity in men (Paolisso et al., 2000; Pieterse et al., 2014). In contrast, a negative association between leptin and sympathetic activity was found in both women and men with a body fat% >25.5%. This is remarkable since body fat% is expected to have a positive correlation with both leptin and sympathetic activity (Charles et al., 2015).

Still, a positive leptin-sympathetic association seems logic from a physiological viewpoint. Leptin stimulates energy-expenditure in fat mass and enhances sympathetic activity in non-thermogenic organs and glands (e.g., kidney and adrenal gland) by an increase in noradrenalin concentrations, the neurotransmitter of the sympathetic nervous system (Vinik et al., 2011). The fact that the effects of leptin are mediated by the sympathetic nervous system, is also supported by a study in animals, where blockage of adrenal activity destroyed the effects of leptin (Vinik et al., 2011).

At follow-up and longitudinally, we found a significant and negative association between leptin and parameters of parasympathetic activity (HF and pNN50) in boys. The association with pNN50 was independent of body fat% and mean heart rate. This association was also found by Flanagan et al. in women and was also reported by Paolisso et al. in men (Paolisso et al., 2000; Flanagan et al., 2007). Our study did not show this significant association in girls. The significantly higher values for pNN50 and HF in boys at follow-up could possibly explain this discrepancy. The inverse association between leptin and the parasympathetic nervous system, also known as the anti-inflammatory component of the autonomic system, is supported by the bi-directional relationship between leptin and inflammation. After all, leptin increases the competence of the immune system and the synthesis of leptin is stimulated by inflammatory cytokines (Jung et al., 2012).

### Association of adiponectin and the autonomic balance

The cross-sectional and longitudinal results confirmed the hypothesis that adiponectin is a positive predictor of parasympathetic activity, but this was only true in girls and after adjustment for heart rate. Contrary, adiponectin was a negative predictor of parasympathetic activity in boys but no longer after adjustment for heart rate.

Studies exploring the association between adiponectin and the autonomic balance in healthy subjects are limited. However, it is expected that the physiological mechanisms in healthy subjects are similar as in patients with metabolic and cardiovascular diseases, though extrapolation of study results of patients into a healthy population should be done with caution. Literature mainly reports a positive association between adiponectin and parasympathetic activity (Fasshauer et al., 2003; Takahashi et al., 2007; Boer-Martins et al., 2011; Barbosa-Ferreira et al., 2015). This positive link between adiponectin and parasympathetic activity was also seen in girls in our cross-sectional and longitudinal analyses but only after correcting for mean heart rate. The results after correction for mean heart rate correspond to what is seen in literature and also seem logic considering the knowledge about adiponectin (lower adiponectin levels as well as lower parasympathetic activity in people with overweight and obesity). Consequently, correction for heart rate seems to have an important influence on the association between HRV and adiponectin and supports the hypothesis that HRV cannot be interpreted correctly without taking heart rate into account (Sacha and Pluta, 2005, 2008; Billman, 2013a).

Based on literature, it is unclear whether adiponectin should be considered as predictor or outcome of HRV. Several studies suggest that sympathetic hyperactivity diminishes the expression of adiponectin in fat tissue (Delporte et al., 2002; Fasshauer et al., 2003; Wakabayashi and Aso, 2004; Takahashi et al., 2007; Hoyda et al., 2009). However, there is doubt whether the low adiponectin concentrations are the cause or consequence of the sympathetic hyperactivity (Takahashi et al., 2007; Hoyda et al., 2009). On the other hand, some studies support the hypothesis that adiponectin is a predictor of HRV. For example, Hoyda et al. described the autonomic effects of adiponectin by depolarization of parvocellular neurons in the paraventricular nucleus and emphasized the importance of adiponectin in the energy and autonomic homeostasis regulation (Hoyda et al., 2009). Our longitudinal analyses confirm adiponectin as predictor of HRV rather than HRV as predictor of adiponectin. Consequently, HRV cannot be used as non-invasive measure for adiponectin levels.

Remarkable in the results of this study is the lower amount of significant findings at baseline compared to follow-up. At first sight, it might be because the follow-up study comprised somewhat less participants. Though, even less associations were significant when analyzing the group of children with both data at baseline and follow up. A possible explanation for this discrepancy could be the difference in age: children were ~2 years older at follow-up. Biological changes in puberty could influence the physiology of the association between adiponectin and the autonomic balance. Possibly, the fat tissue is endocrinologically more active in puberty. This hypothesis is supported by our findings: adiponectin concentrations were significantly higher at follow-up in comparison to baseline, mainly in girls. When comparing boys and girls, significant associations differed as well. As we already suggested for the association between HRV and leptin, this could be due to the significant higher values for pNN50 and HF in boys at follow-up.

### Strengths and limitations

Compared to existing research, our study had several strengths. First of all, the size of the studied cohort (at baseline 249; at follow-up 223 children) is larger than most other published studies. Secondly, this study responds to the lack of studies examining the association between the autonomic balance and the adipokines leptin and adiponectin in healthy people. Especially a study examining this association in children is unique. Another strength is the correction for confounding factors. This study distinguishes itself by correcting for mean heart rate. Splitting the analyses by sex is also an added value of this study. A final strength is the longitudinal analysis to suggest some directionality.

Of course, this study is not without limitations. First of all, results might be difficult to generalize because of a selection bias with more high-educated parents. At baseline, the selected population consisted of less girls and older children than in the total cohort of the ChiBS-study. Because of the exclusion of low-quality data and some technical problems with the measurements, HRV data were not available for all children. Furthermore, LF is no perfect sympathetic parameter as it reflects both sympathetic and parasympathetic activity; and also the traditional interpretation of LF/HF has been criticized (Billman, 2013b). Also, monitoring respiration might be recommended for the frequency domain measures. In the longitudinal analyses, there is the limitation that the leptin analysis kit was different at baseline and at follow-up but a previous study showed that different leptin analysis kits yielded almost indistinguishable concentrations (Carlson et al., 1999).

## Conclusion

In conclusion, high leptin and low adiponectin are unfavorable for the autonomic balance as measured with HRV and consequently for the cardiovascular risk, even during childhood. These associations were independent of body fat%. More research is needed to see whether determination of leptin and adiponectin in blood can be used as a measure of cardiovascular health in overweight and obese children. For example, determination of these biomarkers could be useful in determining the cardiovascular gain and thus in increasing motivation during the follow-up of weight loss. In addition, we suggest to examine whether treatments focusing on high leptin and low adiponectin levels are favorable for someone's cardiovascular health. Those studies could increase the understanding of cardiovascular disease pathogenesis.

## Author contributions

RV has performed the statistical analyses and made a manuscript draft. NM designed the hypothesis, helped in data collection, guided in statistical analyses, and edited the draft.

### Conflict of interest statement

The authors declare that the research was conducted in the absence of any commercial or financial relationships that could be construed as a potential conflict of interest.
