# Supplementary material for: Longitudinal Associations of Leptin and Adiponectin with Heart Rate Variability in Children
Source: Front Physiol. 2017 Jul 12;8:498. doi: 10.3389/fphys.2017.00498 (PMC5506193; doi:10.3389/fphys.2017.00498)
Supplement: Supplementary file 1 [file Table1.DOCX]

**Supplemental tables: RMSSD, SDNN, HFnorm and LFpower.**

**Supplemental table 1: Linear regression with adipokines predicting heart rate variability, 2010 (N=249)**

|  | ♀/♂ | RMSSD | | | SDNN | | | HFnormalized | | | LFpower | | | |
| --- | --- | --- | --- | --- | --- | --- | --- | --- | --- | --- | --- | --- | --- | --- |
|  |  | **adjR²** | **β** | **p** | **adjR²** | **β** | **p** | **adjR²** | **β** | **p** | **adjR²** | **β** | **p** | |
| Leptin | ♂ | -0.006 | -0.051 | 0.576 | -0.001 | -0.082 | 0.364 | 0.006 | 0.118 | 0.190 | 0.001 | -0.096 | 0.286 | |
|  | ♀ | -0.008 | 0.017 | 0.855 | -0.008 | 0.015 | 0.870 | 0.004 | -0.109 | 0.228 | -0.005 | 0.059 | 0.513 | |
| model 1 | ♂ | 0.015 | 0.064 | 0.484 | -0.011 | -0.096 | 0.298 | 0.014 | 0.100 | 0.272 | -0.011 | -0.107 | 0.245 | |
|  | ♀ | -0.014 | 0.023 | 0.804 | -0.007 | 0.025 | 0.788 | 0.006 | -0.133 | 0.150 | 0.009 | 0.085 | 0.356 | |
| model 2 | ♂ | -0.017 | -0.054 | 0.557 | -0.005 | -0.081 | 0.380 | 0.007 | 0.104 | 0.256 | -0.001 | -0.091 | 0.325 | |
|  | ♀ | -0.021 | 0.065 | 0.627 | -0.004 | 0.134 | 0.316 | 0.024 | -0.302 | 0.023 | 0.023 | 0.242 | 0.067 | |
| model 3 | ♂ | -0.023 | -0.054 | 0.561 | -0.012 | -0.081 | 0.383 | -0.002 | 0.104 | 0.258 | -0.010 | -0.091 | 0.327 | |
|  | ♀ | 0.075 | 0.128 | 0.321 | 0.102 | 0.200 | 0.117 | 0.082 | -0.252 | 0.052 | 0.090 | 0.295 | 0.022 | |
| model 4 | ♂ | 0.346 | -0.138 | 0.068 | 0.296 | -0.158 | 0.045 | 0.075 | 0.065 | 0.469 | 0.187 | -0.153 | 0.070 | |
|  | ♀ | 0.398 | -0.047 | 0.661 | 0.397 | 0.033 | 0.757 | 0.197 | -0.358 | 0.004 | 0.211 | 0.186 | 0.128 | |
| Adiponectin | ♂ | -0.001 | -0.082 | 0.362 | -0.007 | -0.038 | 0.676 | 0.010 | 0.135 | 0.132 | -0.008 | -0.020 | 0.826 | |
|  | ♀ | -0.050 | -0.583 | 0.561 | -0.007 | -0.037 | 0.686 | 0.001 | 0.098 | 0.279 | -0.007 | -0.040 | 0.663 | |
| model 1 | ♂ | -0.011 | -0.091 | 0.321 | -0.018 | -0.044 | 0.631 | 0.021 | 0.132 | 0.142 | -0.021 | 0.026 | 0.775 | |
|  | ♀ | -0.012 | -0.045 | 0.625 | -0.007 | -0.026 | 0.779 | -0.004 | 0.089 | 0.331 | 0.002 | -0.025 | 0.784 | |
| model 2 | ♂ | -0.013 | -0.086 | 0.350 | -0.010 | -0.036 | 0.691 | 0.014 | 0.134 | 0.139 | -0.009 | -0.018 | 0.847 | |
|  | ♀ | -0.021 | -0.045 | 0.626 | -0.012 | -0.026 | 0.780 | -0.012 | 0.089 | 0.333 | -0.004 | -0.025 | 0.785 | |
| model 3 | ♂ | -0.019 | -0.079 | 0.393 | -0.017 | -0.031 | 0.742 | 0.007 | 0.138 | 0.132 | -0.018 | -0.018 | 0.847 | |
|  | ♀ | 0.068 | -0.032 | 0.714 | 0.084 | -0.013 | 0.885 | 0.062 | 0.100 | 0.256 | 0.049 | -0.015 | 0.866 | |
| model 4 | ♂ | 0.327 | -0.025 | 0.742 | 0.272 | 0.019 | 0.806 | 0.098 | 0.167 | 0.058 | 0.164 | 0.022 | 0.794 | |
|  | ♀ | 0.398 | -0.037 | 0.599 | 0.396 | -0.017 | 0.806 | 0.148 | 0.098 | 0.246 | 0.196 | -0.018 | 0.823 | |
| ♂ = man -♀ = woman - N = number - RMSSD = root mean square of successive differences – SDNN= standard deviation of all normal RR intervals - LF = low frequency - HF = high frequency - frequency - adjR² = adjusted r² - β= standardized regression coefficient - p = significance – Model 1 = correction for age, socioeconomic status - Model 2 = Model 1 + body fat% - Model 3 = Model 2 + negative emotions - Model 4 = Model 3 + mean heart rate | | | | | | | | | | | | | |  |

| Supplemental table 2: linear regression with adipokines predicting heart rate variability, 2012 (N=223) | | | | | | | | | | | | | |  |
| --- | --- | --- | --- | --- | --- | --- | --- | --- | --- | --- | --- | --- | --- | --- |
|  | **♀/♂** | RMSSD | | | SDNN | | | HFnormalized | | | LFpower | | | |
|  |  | **adjR²** | **β** | **p** | **adjR²** | **β** | **p** | **adjR²** | **β** | **p** | **adjR²** | **β** | **p** | |
| Leptin | ♂ | 0.074 | -0.288 | 0.003 | 0.079 | -0.296 | 0.002 | -0.007 | 0.054 | 0.584 | 0.034 | -0.209 | 0.031 | |
|  | ♀ | -0.008 | -0.026 | 0.780 | -0.007 | -0.043 | 0.646 | -0.006 | 0.052 | 0.576 | -0.009 | 0.000 | 0.999 | |
| model 1 | ♂ | 0.074 | -0.322 | 0.001 | 0.072 | -0.323 | 0.001 | 0.061 | -0.020 | 0.838 | 0.018 | -0.219 | 0.032 | |
|  | ♀ | -0.022 | -0.039 | 0.690 | -0.025 | -0.042 | 0.674 | -0.020 | 0.037 | 0.704 | -0.022 | 0.011 | 0.911 | |
| model 2 | ♂ | 0.067 | -0.274 | 0.058 | 0.069 | -0.236 | 0.100 | 0.059 | 0.074 | 0.606 | 0.014 | -0.133 | 0.367 | |
|  | ♀ | 0.021 | 0.244 | 0.112 | 0.021 | 0.249 | 0.105 | -0.025 | 0.117 | 0.453 | 0.039 | 0.341 | 0.026 | |
| model 3 | ♂ | 0.061 | -0.259 | 0.076 | 0.059 | -0.234 | 0.109 | 0.050 | 0.068 | 0.643 | 0.007 | -0.121 | 0.416 | |
|  | ♀ | 0.012 | 0.244 | 0.114 | 0.012 | 0.250 | 0.105 | -0.024 | 0.113 | 0.470 | 0.038 | 0.345 | 0.024 | |
| model 4 | ♂ | 0.052 | -0.258 | 0.078 | 0.050 | -0.235 | 0.109 | 0.084 | 0.077 | 0.589 | 0.001 | -0.125 | 0.406 | |
|  | ♀ | 0.032 | 0.232 | 0.129 | 0.044 | 0.236 | 0.121 | -0.019 | 0.121 | 0.438 | 0.046 | 0.336 | 0.028 | |
| model 5’ | ♂ | 0.434 | -0.071 | 0.535 | 0.389 | -0.057 | 0.635 | 0.082 | 0.130 | 0.376 | 0.151 | -0.002 | 0.989 | |
|  | ♀ | 0.488 | 0.178 | 0.109 | 0.416 | 0.189 | 0.111 | 0.071 | 0.082 | 0.581 | 0.250 | 0.300 | 0.027 | |
| Adiponectin | ♂ | 0.035 | -0.211 | 0.029 | 0.047 | -0.237 | 0.014 | 0.003 | -0.110 | 0.259 | 0.028 | -0.193 | 0.046 | |
|  | ♀ | 0.002 | 0.105 | 0.264 | 0.005 | 0.118 | 0.207 | -0.007 | 0.047 | 0.620 | 0.002 | 0.102 | 0.274 | |
| model 1 | ♂ | 0.021 | -0.215 | 0.030 | 0.030 | -0.240 | 0.015 | 0.077 | -0.127 | 0.185 | 0.010 | -0.195 | 0.050 | |
|  | ♀ | -0.011 | 0.110 | 0.254 | -0.012 | 0.117 | 0.223 | -0.018 | 0.054 | 0.575 | -0.013 | 0.098 | 0.308 | |
| model 2 | ♂ | 0.069 | -0.191 | 0.049 | 0.089 | -0.213 | 0.027 | 0.071 | -0.120 | 0.212 | 0.037 | -0.175 | 0.075 | |
|  | ♀ | 0.008 | 0.100 | 0.292 | 0.008 | 0.108 | 0.259 | -0.028 | 0.053 | 0.581 | 0.002 | 0.090 | 0.348 | |
| model 3 | ♂ | 0.075 | -0.211 | 0.032 | 0.085 | -0.224 | 0.022 | 0.062 | -0.117 | 0.231 | 0.036 | -0.190 | 0.057 | |
|  | ♀ | -0.001 | 0.102 | 0.290 | -0.001 | 0.107 | 0.268 | -0.025 | 0.065 | 0.504 | -0.002 | 0.082 | 0.395 | |
| model 5 | ♂ | 0.434 | -0.054 | 0.493 | 0.394 | -0.078 | 0.336 | 0.080 | -0.074 | 0.458 | 0.160 | -0.096 | 0.319 | |
|  | ♀ | 0.509 | 0.183 | 0.008 | 0.436 | 0.181 | 0.014 | 0.079 | 0.103 | 0.269 | 0.234 | 0.137 | 0.107 | |
| ♂ = man -♀ = woman - N = number - RMSSD = root mean square of successive differences – SDNN= standard deviation of all normal RR intervals - LF = low frequency - HF = high frequency - LF/HF = low frequency/high frequency - adjR² = adjusted r² - β= standardized regression coefficient - p = significance – Model 1 = correction for age, socioeconomic status - Model 2 = Model 1 + body fat% - Model 3 = Model 2 + negative emotions - Model 4 = Model 3 + pubertal status (only relevant for leptin)- Model 5’ (for leptin)= Model 4 + mean heart rate - Model 5 = Model 3 + mean heart rate | | | | | | | | | | | | | |  |

| Supplemental table 3: Mixed model regression, longitudinally | | | | | | | | | |  |
| --- | --- | --- | --- | --- | --- | --- | --- | --- | --- | --- |
|  | **♀/♂** | RMSSD | | SDNN | | HFnormalized | | LFpower | | |
|  |  | **b** | **p** | **b** | **p** | **b** | **p** | **b** | **p** | |
| Leptin |  |  |  |  |  |  |  |  |  | |
| model 1 | ♂ | -4.017 | 0.010 | -0.003 | 0.020 | 0.158 | 0.790 | -32.877 | 0.131 | |
|  | ♀ | -0.421 | 0.612 | -0.000 | 0.720 | 0.390 | 0.328 | -0.937 | 0.930 | |
| model 2 | ♂ | -2.920 | 0.103 | -0.001 | 0.241 | 0.417 | 0.550 | -14.120 | 0.563 | |
|  | ♀ | -0.214 | 0.798 | -1.421E^-5^ | 0.981 | 0.337 | 0.408 | 2.184 | 0.834 | |
| model 3 | ♂ | -1.013 | 0.498 | -0.000 | 0.816 | 0.896 | 0.243 | 1.718 | 0.944 | |
|  | ♀ | 0.338 | 0.565 | -0.000 | 0.294 | -0.293 | 0.500 | 12.817 | 0.208 | |
| Adiponectin |  |  |  |  |  |  |  |  |  | |
| model 1 | ♂ | -1.954 | 0.011 | -0.001 | 0.007 | -0.305 | 0.291 | -21.900 | 0.041 | |
|  | ♀ | 1.437 | 0.074 | 0.001 | 0.030 | 0.134 | 0.690 | 21.394 | 0.043 | |
| model 2 | ♂ | -1.771 | 0.019 | -0.001 | 0.014 | -0.302 | 0.299 | -19.881 | 0.062 | |
|  | ♀ | 1.406 | 0.076 | 0.001 | 0.031 | 0.130 | 0.699 | 21.098 | 0.043 | |
| model 3 | ♂ | -0.413 | 0.494 | -0.000 | 0.349 | -0.078 | 0.790 | -7.748 | 0.437 | |
|  | ♀ | 1.722 | 0.001 | 0.001 | 0.001 | 0.216 | 0.498 | 23.354 | 0.012 | |
| ♂ = man -♀ = woman - N = number - RMSSD = root mean square of successive differences – SDNN= standard deviation of all normal RR intervals - LF = low frequency - HF = high frequency - LF/HF = low frequency/high frequency - b= regression coefficient of the time*predictor parameter to show the longitudinal relation- p = significance - Model 1 = correction for age, socioeconomic status - Model 2 = Model 1 + body fat% - Model 3 = Model 2 + mean heart rate | | | | | | | | | |  |
